# Department of Error

**DOI:** 10.1016/S0140-6736(19)32010-0

**Published:** 2019-08-31

*The Pneumonia Etiology Research for Child Health (PERCH) Study Group. Causes of severe pneumonia requiring hospital admission in children without HIV infection from Africa and Asia: the PERCH multi-country case-control study.* Lancet *2019;* **394:** *757–79—*In this Article, the list of contributors to the study design was incorrect. David R Murdoch contributed to designing the study, not David P Moore. This correction has been made to the online version as of Aug 29, 2019, and the printed version is correct.

